# Recurrent Venous Thromboembolism: What Is the Risk and How to Prevent It

**DOI:** 10.6064/2012/391734

**Published:** 2012-09-17

**Authors:** Gualtiero Palareti

**Affiliations:** Department of Angiology and Blood Coagulation, Via Albertoni 15, 40138 Bologna (BO), Italy

## Abstract

Venous thromboembolism (VTE) that includes deep vein thrombosis and/or pulmonary embolism is a frequent, severe, and potentially lethal disease. After a first episode, VTE has a strong tendency to recur. While VTE is an acute disease, it may have variable outcomes in early and late phases after initial presentation. Furthermore, the incidence of late, clinically important consequences (postthrombotic syndrome and/or chronic thromboembolic pulmonary hypertension) increases in case of recurrent events. The aims of the present review are (i) to analyze the incidence and risk factors for recurrence of VTE (either those related to the type of first thrombotic event or to the patients), the risks associated with occurrence of recurrent events, and the problems linked to the diagnosis, not always easy, of recurrent events; (ii) to discuss whether or not it is possible to predict the individual risk of recurrence after a first event, by stratifying patients at high or low risk of recurrence, and how this can influence their treatment; (iii) to comment what the current guidelines and guidance suggest/recommend about anticoagulant treatment after a first VTE event and, finally, to propose practical indications on how to manage individual patients affected by VTE.

## 1. Introduction

Venous thromboembolism (VTE), that includes deep vein thrombosis (DVT) and/or pulmonary embolism (PE), is a frequent, severe, potentially lethal, yet treatable, and acute disease. Its incidence in developed countries is high, affecting from 1 to 2 per 1000 persons per year [1–7]. VTE is the third most common cardiovascular disease, after myocardial infarction and stroke [7]. While VTE is an acute disease, it may have variable outcomes in early and late phases after initial presentation. After a first episode, VTE has a strong tendency to recur. Though the current standard therapy, based on immediate anticoagulation with heparin or derivatives followed by vitamin K antagonists (VKAs), is very effective [8], recurrent VTE episodes or extension of the disease occur in a nonnegligible portion of patients (*≈*4% after a DVT), even in the presence of adequate therapy [9–11]. Furthermore, the benefit of anticoagulation is lost after discontinuation [12]. 

VTEs also have late, clinically important consequences. A late and frequent outcome of DVT is the occurrence of postthrombotic syndrome (PTS), a condition that greatly affects the morbidity and quality of life of patients and leads to high social costs. In a cohort of patients with DVT followed for two years, mild, moderate, or severe signs of PTS were detected in 30%, 10%, and 3%, respectively [13]. Chronic thromboembolic pulmonary hypertension was detected in as many as 3.8% of patients two years after an acute episode of PE [14]. All these results seem to justify why some authors claim VTE as a chronic disease [15]. 

The aims of the present review are (i) to analyze the incidence and risk factors for recurrence of VTE, including DVT of lower limbs and PE, (ii) to discuss how to stratify patients at high or low risk of recurrence and how this can influence their treatment, and (iii) to propose a way to manage individual patients affected by VTE. 

## 2. Incidence of VTE Recurrence

 Data on incidence of recurrent VTE can be found in several available epidemiological and cohort studies, as well as randomized clinical trials. The risk of VTE recurrence is present even during anticoagulant therapy. An overall rate of 5.5% of recurrent VTE during the initial 3 months of anticoagulant treatment was found in one study [11] and of 7.0% in the very large cohort of patients included in the RIETE registry [16]; in the latter study patients with recurrence during VKA treatment were more frequently males, younger, and with a higher incidence of cancer than those without recurrence [16]. 

The incidence of recurrence is obviously higher in cases of long followup, and especially after VKA treatment is interrupted. Hansson et al. [17] followed a cohort of patients with a first DVT and reported a 5-year cumulative incidence of recurrent VTE of 21.5% after a first DVT and of 27.9% after a second DVT. Heit et al. [15] followed 1719 patients from the Olmsted County cohort and found a cumulative incidence of first VTE recurrence of 30.4% at ten years. The hazard rate of recurrence was highest in the first 6–12 months after the initial event but never fell to zero, thus suggesting that VTE is a chronic disease with episodic recurrence. Prandoni et al. [18] followed a cohort of 1626 patients with first VTE for up to 10 years. After a median followup of 50 months recurrent VTE occurred in 22.9% of patients, the cumulative incidence of recurrent VTE was 11.0%, 19.6%, 29.1,% and 39.9% after 1, 3, 5 and 10 years, respectively. The cumulative incidence of recurrence was *≈*16% after a mean followup of 1216 days from the initial VTE event in the patients included in the Worcester VTE study [19]. An incidence of recurrence of 26.1% at 5 years was recorded in a small series of patients with DVT by Labropoulos et al. [20]. Poli et al. [21] have prospectively followed a series of 206 patients with acute PE. After interruption of VKA treatment a recurrent VTE event occurred in 11.2% of patients during a median period time of 19 months (range 1–120 months); the recurrent event was a new PE episode in 48% of cases. 

 Few data are available on the rate of recurrent VTE in non-Caucasian populations. A recent population-based cohort study in the Taiwanese population found a recurrent rate of recurrence of 5.1% person-years with a cumulative rate of 14.4% after 47 months of followup, a rate that is, as expected, lower than in the Caucasian population [22]. 

 As regards the incidence of recurrence in patients included in clinical trials, Schulman et al. [23] randomized patients with a first VTE to receive anticoagulant treatment for 6 weeks or 6 months and after 2 years of followup recurrence occurred in 18.1% and 9.5% of patients, respectively. Pinede et al. [24] randomized patients with VTE to receive 3 or 6 months of anticoagulation and after 15 months found no difference in recurrences (6.4% and 7.4%, resp.). 

 The incidence of recurrent VTE is high during the first years following the index episode and declines thereafter [25].

## 3. Diagnosis of Recurrences and How They Usually Present

 An unambiguous definition of recurrent VTE is the one proposed by Heit et al. [26] in which recurrence was defined “as venous thrombosis of a site that was either previously uninvolved or had interval documentation of incident DVT or PE resolution.” More uncertain is the idea of including among recurrences cases with extension of a previously diagnosed thrombotic process. 

Symptoms and/or signs that can be attributable to recurrent VTE occur frequently during the natural history of the previous event. This is why diagnosis of recurrent VTE should be based on objective assessments (for review see Labropoulos et al. [27]). As regards the workup for diagnosis/exclusion of suspected recurrent DVT, Prandoni et al. proposed a simple ultrasound method for measuring thrombus regression in patients with previous proximal DVT; the vein diameter under maximum compression (thrombus thickness) is measured in the involved venous segment and a noncompressibility of a previous normal(ized) venous segment or an increase of the residual diameter of ≥2 mm is considered diagnostic of recurrent proximal DVT [28]. The same group later proposed to add D dimer in the diagnostic workup for recurrent DVT and increase up to 4 mm the diagnostic limit of the difference in residual diameter; they concluded that it is safe to exclude recurrent proximal DVT in cases with negative D dimer and stable or slightly increased (≤4 mm) residual vein diameter [29]. Other authors investigated how the change in thrombus length measured by ultrasound may be relevant for diagnosis of recurrence and found that an increase in thrombus length of 9 cm or more is supportive of recurrent DVT, whereas an increase <9 cm can be within the limits of measurement errors [30]. 

There is unanimous consensus that to help the diagnostic workup for suspected recurrent VTE a baseline imaging of leg veins and/or pulmonary arteries at the moment anticoagulant therapy is completed would be highly recommended, especially in those patients with an unprovoked VTE, who are at higher risk of recurrence. A recent clinical study confirmed that available baseline imaging was of substantial help in interpreting diagnostic tests in cases of suspected recurrent VTE [31].

Recurrences are usually diagnosed because of the appearance of new symptoms, but can also be incidentally detected during periodical examinations. Recurrences may affect the ipsi- or contralateral leg or present as symptomatic PE. In contrast with what might be expected, the risk of a recurrent event in the contralateral leg has been reported significantly higher than in the ipsilateral leg (1.6; CI 1.4–1.9) [32]. Patients with a first symptomatic PE have a higher risk of recurrence than those with DVT and are also at higher risk of symptomatic PE at recurrence [33]. It has been shown that patients presenting with PE have three times higher risk of PE recurrence than patients presenting with DVT [34].

## 4. The Clinical Risks of Recurrent VTE

 Recurrent DVT in the same leg is a strong risk factor for development of PTS (HR 6.4; CI 3.1–13.3 [35]). Recurrent PE is the strongest risk factor for pulmonary hypertension (OR 19.0 CI 4.5–79.8 [36]). A prospective study showed that survival from recurrent VTE after 8 years was 70.2% (CI 64.7–75.6) [35]; similar results were obtained by the same group a decade later in a large series of patients with 10 years of follow-up [18]. A recent systematic review [37] reported that during the first 3 months of anticoagulant therapy the rate of fatal recurrent VTE was 0.4% (CI 0.3–0.6), and the case-fatality rate of recurrent VTE was 11.3% (CI 8.0–15.2); after completion of anticoagulation, the rate of fatal recurrences was 0.3% patient-years (CI 0.1–0.4), and the case-fatality rate was 3.6% (CI 1.9–5.7). 

The higher risk of recurrent PE after a first symptomatic PE has been advocated as a factor that can significantly increase the risk of mortality in patients with a first PE [38]. However, in a recent retrospective study that examined the data of a large series of young patients (<55 years) after an initial episode of symptomatic PE, it was found that a recurrent event occurred in only 12% of patients over a mean period of 3.2 years and that case-fatality rate was extremely low (0.16% per year starting at six months after the index event) [39]. These data are in agreement with the annual rate of PE-related deaths (0.19%) observed in a recent inception cohort of patients with a first episode of symptomatic VTE who discontinued anticoagulant therapy [40].

## 5. Risk Factors for Recurrence

 A large number of studies have investigated factors or conditions that may play a possible role as risk factors in recurrence; these factors may be related either to the index thrombotic event itself or to the patients (Table 1).

### 5.1. Unprovoked (Idiopathic) or Secondary (Provoked) First VTE Event

The most important factors that influence the risk of recurrent VTE after stopping VKA are (a) presence of temporal association with reversible risk factors (secondary or provoked event), (b) absence of any apparent clinical risk factors (unprovoked or idiopathic), and (c) presence of a persistent clinical factor, such as active cancer or antiphospholipid syndrome. Many randomized clinical trials and observational cohort studies have unanimously concluded that the risk of recurrence is much higher after unprovoked than after secondary events [17, 23, 24, 35, 41–47]. Prandoni et al. in a large cohort of patients found that after 10 years of followup the cumulative incidence of recurrence was about 50% in patients with unprovoked events and less than half that rate in those with secondary events [18]. In a recent systematic review of available studies Iorio et al. calculated that, among patients with VTE provoked by a reversible factor, the risk of recurrence was much lower if the provoking factor was recent surgery (0.7% patient-year) compared with a nonsurgical factor (e.g., estrogen therapy, pregnancy, leg injury, and long flight; (4.2% patient-year)) [48]. 

Patients with a persistent risk factor for VTE have a high risk of recurrence. It is known that the risk of recurrence if anticoagulation is stopped is very high in patients with active cancer, especially if with metastatic disease or during concurrent chemotherapy [43, 49]; an annual risk of 15% in these patients has been estimated [50]. A high risk of recurrence is also present in patients with antiphospholipid syndrome [51–53]. This is why in the above clinical conditions the interruption of anticoagulant treatment is not recommended.

### 5.2. Site and Extension of VTE

 It has been shown that during the initial 3 months of anticoagulant therapy patients with more proximal (iliofemoral) vein thrombosis have a higher risk of recurrence (11.8%) than those with popliteal or femoral DVT (*≈*5%) [11]. After discontinuation of secondary thromboprophylaxis a higher rate of recurrent event has been reported in patients whose index event was symptomatic PE (17.3%) than in those with DVT without symptoms of PE (9.5%) [33]. These results were, however, not confirmed in more recent studies. A relative risk of recurrent VTE of 2.1 (CI 1.2–3.7) for isolated DVT versus isolated PE was found by Kovacs et al. [54]. A patient-level meta-analysis involving more than 2500 patients from various prospective studies calculated a 5-year cumulative rate of recurrent VTE of 22.6% in patients with PE and of 26.4% in those with proximal DVT [34]. However, the clinical risk associated with the recurrent event was not the same in the two presentations since, as already mentioned, the recurrence as PE was 3-times higher in patients presenting with PE than in those presenting with proximal DVT. 

Thromboses limited to deep calf veins (distal DVT) are associated with a lower risk of recurrence than proximal DVT or PE [23, 24, 55]. The rate of recurrent VTE has been reported to be 4.8-times higher in patients with proximal versus isolated distal DVT [34]. It has been shown that while the rate of recurrence was similar in cases with unilateral or bilateral proximal DVT, it was lower in cases with unilateral (7.7%) versus bilateral (13.3%) distal DVT.

### 5.3. Gender and Age

 Several studies have unanimously shown that the risk of recurrence is higher in men than in women [56–59]. Relative risk of recurrent VTE in men versus women was 1.6 (CI 1.2–2.0) in a meta-analysis that examined more than 5000 patients from 15 studies [60]. A more recent patient-level meta-analysis of seven prospective studies calculated a three-year incidence of recurrence of 9.1% (7.3% to 11.3%) in women and 19.7% (16.5% to 23.4%) in men. The risk of recurrence remained higher in men (HR 1.8, 1.4 to 2.5) after adjustment for women with hormone-associated initial VTE; it was higher in men (HR 2.2, CI 1.7 to 2.8) when unprovoked VTE was considered, but was not different versus women when analysis was limited to VTE occurring after exposure to a major risk factor [61]. For a review on this issue see [62].

 The evidence that the risk of a first VTE increases exponentially with age is unquestionable. More conflicting are data on the effect of age on the risk of recurrent VTE. An increased risk associated with age at first event was found in only some [18, 43, 63], though not in other studies [17, 59, 64, 65]. In contrast, an analysis of randomized controlled studies concluded that older age was associated with a decreased risk of VTE recurrence [66]. It is of interest to note that a higher than expected risk of recurrent VTE, stable over many years, has been shown in a large series of young women (<45 years age), a population usually believed to be at low risk [67].

### 5.4. Thrombophilia

In recent decades several coagulation abnormalities, leading to loss of anticoagulant function or gain of procoagulant activity, have been shown to be associated with an increased risk of thrombosis. They include deficiencies of the natural anticoagulants antithrombin, protein C, and protein S (rather rare alterations), the very frequent factor V Leiden or G20210A prothrombin gene mutations, and high levels of factors VIII, IX, or XI (for reviews see [68, 69]. The presence of one or more of these alterations, that in most cases are hereditary, is termed “thrombophilia.” The presence of thrombophilia as risk factor for the first VTE episode is undisputed; its impact on the recurrence rate is, on the contrary, still a highly debated issue and the several clinical studies that focused on this yelded inconsistent results. It has been shown that single or multiple thrombophilic defects are not associated with a higher rate of recurrent VTE during VKA treatment [70]. It has recently been reported that the risk of recurrent VTE was higher in patients with hereditary deficiency of natural anticoagulants (protein S, protein C, or antithrombin), a condition present in less than 10% of VTE patients [71], after a spontaneous (relative risk 1.5 (0.95–2.3) but not after a secondary first event [72].

Few studies found that carriers of heterozygous factor V Leiden or prothrombin mutation were at higher risk of VTE recurrence [73, 74], while most did not find any significant difference in the risk between carriers and noncarriers [45, 75–77]. Carriers of both factor V Leiden and the G20210A prothrombin mutation were reported to have an increased risk of recurrent DVT and were proposed for lifelong anticoagulation [78]. However, more recently individuals with homozygous factor V Leiden and/or homozygous prothrombin G20210A or double heterozygous carriers of both alterations were not shown to have a higher risk of recurrent VTE [79].

Interestingly, a higher rate of recurrence was reported in two studies in subjects with thrombophilic alteration [80] factor V Leiden or prothrombin mutation [81] who had received a short period of anticoagulation (3 months) but not in those treated for longer periods.

A higher rate of recurrence has been shown by several authors in subjects with high plasma levels of factor VIII [82–84] and factor IX [85] though others found no impact on the risk of recurrence of high factor VIII, IX, and XI, either alone or in association with heterozygous factor V Leiden [86]. 

Abnormally short activated partial thromboplastin time (APTT) values were found to be associated with increased risk of recurrent VTE after anticoagulation interruption, a test that may be helpful for stratifying high or low risk of recurrence [87, 88].

An increased risk of recurrent VTE has been reported in subjects with high levels of thrombin-activable fibrinolysis inhibitor (TAFI) [89], low vitamin B6 levels [90], low levels of free tissue factor pathway inhibitor (TFPI) [91], and elevated albuminuria [92] while high levels of apolipoprotein A1 and HDL have not been reported as being associated with lower risk of recurrence [93].

### 5.5. Duration, Intensity, and Quality of Anticoagulant Treatment for the First VTE

In recent decades many clinical trials have compared different time-limited durations of anticoagulation with VKA (adjusted to maintain the INR between 2.0 and 3.0) with the primary goal of identifying the optimal duration of therapy, that is, the shortest period of anticoagulation to achieve the lowest posttreatment risk of recurrence, balancing this risk with that of major bleeding associated with anticoagulation. Several reviews [94–96], meta-analyses, and systematic reviews of the literature [97–100] have focused on this issue.

Some randomized trials assessed whether very short periods of treatment (<3 months) might be sufficient [23, 41, 101]. All these studies concluded that anticoagulation should last at least 3 months since shorter periods are inadequate, the only possible exception being that of isolated distal DVTs if caused by a major transient risk factor that can be treated for 6 weeks [23]. 

Other studies compared 3 months of VKA treatment with longer courses (between 6 months and 2 years) for patients with idiopathic VTE and found that the rate of recurrence during treatment was extremely low (about 1%), while it was about 15% during followup in the patients who stopped anticoagulation after 3 months [24, 102]. Agnelli et al., in two subsequent studies on patients with idiopathic DVT [12] or PE [103], randomized to receive 3 months or one year of VKA, found that during a long-term followup after anticoagulation discontinuation the incidence of recurrent VTE was similar in both groups of patients. 

These results indicate that (i) VKA therapy is highly effective in protecting against recurrent VTE during treatment, (ii) treatment should be given for no less than 3 months (probably 6 months for patients with thrombophilic abnormalities [80, 81]), and (iii) prolonging treatment beyond that recommended period delays recurrence but does not reduce the risk of recurrence after treatment is stopped. This is the main reason why indefinite anticoagulation after a first episode of unprovoked VTE was advocated [104] and highly recommended in the recently published 9th edition of the Antithrombotic Therapy and Prevention of Thrombosis: ACCP Guidelines, provided that patients are at low risk of bleeding and can undergo well-managed VKA treatment [50].

 To enhance the benefit/risk relationship of chronic anticoagulation in patients with VTE and to improve the quality of life of patients, the use of a lower therapeutic range (1.5–2.0 INR) after an initial period of standard intensity VKA treatment (2.0-3.0 INR) was evaluated in two randomized trials. In one study, after full-dose anticoagulation therapy for a median of 6.5-month patients with idiopathic VTE was randomized to receive placebo or low-intensity warfarin, with the rates of recurrence being 7.2% person-years and 2.6% in the two groups, respectively, without differences for major bleeding complications [105]. In the second study, after 3 or more months of standard VKA therapy, patients were randomized to receive in double-blind fashion low (1.5–1.9 INR) or conventional (2.0-3.0 INR) intensity VKA therapy; at the end of an average followup of 2.4 years the rates of recurrent VTE were 1.9% person-years and 0.7% in the two groups, respectively, without significant differences in the incidence of major and overall bleeding events [106].

The quality of anticoagulant treatment, including both initial heparin and subsequent VKA therapies, has been investigated as risk factors for recurrence of VTE during treatment and also after anticoagulation discontinuation. Though initial studies found an association between an early subtherapeutic APTT (to regulate heparin therapy) and VTE recurrence [107], these observations were not confirmed by subsequent meta-analyses of randomized trials [108]. A population-based inception cohort study, that involved residents of the Olmsted County, MN, USA, has found that a greater proportion of time spent on heparin, but at a lower anticoagulant intensity, and of time on warfarin with an INR ≥ 2.0 were factors associated with a significantly reduced hazard of VTE recurrence [26]. It was also found that poor warfarin anticoagulation quality (more time spent at INR < 1.5) during the first 3 months after unprovoked VTE was a long-term risk factor for recurrence of VTE after VKA therapy interruption [109]. These results were confirmed by some [110] though not all subsequent studies [111, 112].

### 5.6. Pregnancy and Contraceptive/Hormone Replacement Therapy

It is well known that pregnancy and especially puerperium are conditions carrying a higher risk of VTE. VTE risk increases 4-fold during pregnancy and 20-fold during puerperium [113]. More conflicting are data on the risk of recurrence during pregnancy in women who had a previous VTE and especially in those with pregnancy-associated VTE (for a review see James et al. [114]). This issue is particularly important from a clinical point of view since it refers to the question whether or not women with previous thrombosis should receive prophylaxis during pregnancy and after delivery. 

In a prospective study in women with previous VTE, Brill-Edwards et al. [115] found a low risk for recurrent antepartum VTE despite withholding prophylactic anticoagulation, and they concluded that antepartum prophylaxis should be considered only in patients with idiopathic thrombosis and those with thrombophilia.

Other studies have provided different results. In a retrospective study it was found that pregnancy leads to a temporary and a more than 3-fold increase in the risk of symptomatic recurrent thrombosis [116]. Another, more recent, retrospective study [117] found that the cumulative rate of recurrence was lower in women with pregnancy-associated index VTE than in women with unprovoked VTE (5.8% versus 10.4%; *P* = 0.02). However, the incidence of recurrence during subsequent pregnancies was higher in the pregnancy group (4.5%) than in the unprovoked group (2.7%; RR = 1.7, CI: 1.0–2.8). The authors conclude that women with pregnancy-associated VTE have a significantly lower long-term risk of recurrent VTE versus women with unprovoked VTE, but a higher risk of recurrent VTE during a subsequent pregnancy. These data suggest that prophylactic administration of LMWH during pregnancy and puerperium might reduce the risk of recurrent pregnancy-associated VTE.

Combined oral contraceptives and hormone replacement therapy increase the risk of VTE, but data on risk of recurrence in subjects with previous hormone-related VTE are sparse, and the risk has not been precisely determined. This issue is relevant for the problem of whether women with hormone-related VTE, should be classified as having “unprovoked” or “provoked” VTE, and therefore has great clinical importance since it influences the decision about the secondary prevention and duration of anticoagulation. Since most clinical studies have not found significant differences in the risk of VTE recurrence in women with hormone-related or idiopathic first VTE we agree that hormone-related VTE should not be considered as a provoked event, and other risk factors for recurrence should be considered to decide the length of anticoagulant therapy for secondary prevention. In fact, Kyrle et al. [56] reported a cumulative probability of recurrence at five years after followup in 5.9% (CI 0.6–11.1) of women with a hormone-related first thrombosis and 4.3% (CI 0 to 10.1; *P* = 0.8) in women of the same age with a first idiopathic event. Baglin et al. found that women exposed to hormones (primarily oral contraceptives) were not at lower risk of recurrence than other women [57]. Similar results were reported by Le Gal et al., analyzing the data from the REVERSE cohort study, found that the risk of recurrent VTE was low in women after a first otherwise unprovoked estrogen-associated VTE, but not significantly lower than in women whose VTE was not related with hormone treatments [118]. Some different results were reported by Cushman et al. who, in a posthoc analysis of the PREVENT study, found that much of the lower rate of VTE recurrence recorded in women versus men was explained by a lower recurrence risk among women with hormone-related thrombosis (46% lower risk than other women) [119].

A retrospective cohort study investigated the risk factors for recurrence after a first VTE occurring in women taking oral contraceptives [120]. The cumulative incidence of recurrent VTE in women who had stopped anticoagulation was 5.1% and 14.2% after 1 and 5 years, respectively. Significant factors associated with recurrence were renewed use of contraceptives (HR = 8.2 (2.1–32.2)), antiphospholipid syndrome (HR = 4.1 (1.3–12.5)), protein C deficiency, and factor II G20210A (HR = 2.7 (1.1–7)). The high risk of recurrence due to hormonal therapy was also found in a study that randomized females with previous VTE to receive hormone replacement therapy or placebo [121]. The study was terminated prematurely because the incidence of recurrence was 10.7% and 2.3% in the groups receiving hormone therapy and placebo, respectively.

### 5.7. Persistence of Residual Vein Obstruction

Several studies evaluated thrombus regression by compression ultrasonography after symptomatic leg proximal DVT reporting normalization rates at 1 year or later ranging from 36% to 96% of patients [122–126]. In 2002 two prospective clinical studies [127, 128] assessed the value of persistence of residual vein thrombosis as risk factor for recurrent VTE. The ultrasonography criteria for residual thrombus were different in the two studies: present when residual thrombus occupied, at maximum compressibility, more than 40% of the vein area calculated in the absence of compression [127], or when vein diameter during maximal compression was 2 or 3 mm or more in a single or in two consecutive tests [128]. Notwithstanding these differences, both studies consistently found that the thrombus persistence was an important risk factor favoring recurrence of a new thrombosis and might be a relevant tool to determine the optimal duration of anticoagulation. Prandoni et al. found a hazard ratio for recurrence of 2.4 (CI 1.3–4.4) [128] and Young et al. of 2.2 (CI 1.19–4.21) [129]. Some studies confirmed the predictive value of residual vein obstruction for VTE recurrence in patients with unprovoked events [130, 131] and one in patients with cancer [132], while others did not [133–136]. Two randomized studies showed that persistent residual thrombus was a good criterion for determining the duration of anticoagulation [137, 138]. Finally, three systematic reviews or meta-analyses focused on this issue but led to nonconsistent conclusions; two showed a positive relationship between residual thrombosis and recurrent VTE [139, 140], while the other [141] concluded that the association between residual vein obstruction and recurrent VTE was only modest and that the presence of a residual thrombus did not seem to be a predictor of recurrent VTE in patients with unprovoked DVT. 

### 5.8. Cancer

Three cohort studies reported a markedly higher risk of recurrent VTE, even during VKA anticoagulation in patients with cancer [49, 142, 143]. A high hazard ratio for recurrence was also found by Heit et al. in patients with cancer, in particular in those who were receiving chemotherapy [43]. An increased risk of recurrent VTE was found in patients with malignancy after development of new metastases [144]. A more recent study, analyzing a large series of patients included in the RIETE registry, found that during the first 3 months of VKA treatment, recurrent VTE occurred in 1.4% of patients without cancer, 6.6% with distant metastases and 3.2% with more limited disease; the odds ratio for recurrent VTE in the whole group of cancer patients in comparison to patients without malignancy was 3.2 (CI, 2.4–4.3), but it was 5.2 (3.4–7.9) in patients with metastases [145].

### 5.9. Other Conditions/Factors

*Obesity* is a risk factor for first VTE. Few and nonconsistent data are available on its value as risk factor for VTE recurrence. Romualdi et al. followed a cohort of patients with first DVT with or without abdominal obesity (waist circumference >102 cm in men and >88 cm in women) [146]; recurrent DVT was documented in 27.6% of patients with and 31.0% without abdominal obesity (HR 1.26; 0.47–3.4). Conversely, Eichinger et al. [147], in a large prospective cohort of patients with first VTE followed after anticoagulation interruption, found that BMI was significantly higher in those with recurrence and that the probability of recurrence at 4 years was 9.3% (CI 6.0–12.7) in patients of normal weight and 16.7% (CI 11.0–22.3) and 17.5% (CI 13.0–22.0) in overweight or obese, respectively. They concluded that excess body weight is a risk factor for recurrent VTE.

It has been shown that the presence of an *inferior vena cava filter*, while reducing the subsequent risk of PE, increases that of DVT [148]; in that study, the cumulative incidence of recurrent VTE at 5 years in patients with cava filters was 26%. A population-based study showed that filter placement was not associated with a significant reduction in the 1-year incidence of rehospitalization for PE and was associated with a significantly higher relative hazard of rehospitalization for DVT in patients who initially manifested PE [149]. A recent prospective study of a large series of patients with retrievable cava filters showed a cumulative rate of recurrent events of 24.1% at 18 months [150].

Frequent recurrent thrombosis was found in a large multicenter cohort of patients affected by *polycythemia vera and essential thrombocythemia*, with an incidence of 7.6% patient-years [151]. The presence of homozygous JAK2 V617F mutation in patients with essential thrombocythemia is an independent risk factor for recurrent thrombosis; it has been shown in fact that carriers have an increased risk of recurrence versus wild-type patients (HR 6.15, CI 1.51–24.92) [152].

## 6. The Value of D Dimer as a Risk Factor for VTE Recurrence 

### 6.1. D Dimer Measured Postanticoagulation

D dimer is a product of lysis of stabilized fibrin clot considered an indirect marker of coagulation activation. In 2002 Palareti et al. [44] in a prospective inception cohort of patients with previous first VTE repeatedly measured D dimer levels during anticoagulation and after its discontinuation. Abnormal D dimer levels were observed in 15.5% of patients on the day of VKA discontinuation and in 40.3% and 46.2% after 1 and 3 months, respectively. The hazard ratio (HR) for recurrence was significantly higher in subjects with abnormal versus normal D dimer at 3 months (HR 2.45; CI 1.28–4.53; *P* < 0.01). The same group in a subsequent study investigated the predictive value for recurrent VTE of D dimer levels measured 1 month after anticoagulation interruption in patients with a previous unprovoked event, including both carriers and noncarriers of congenital thrombophilia [153]. Altered D dimer results were associated with a significantly higher recurrence rate in patients with an unprovoked qualifying VTE event; the significant association was confirmed by multivariate analysis after adjustment for other risk factors. Similar results were obtained in subsequent cohort studies [134, 154–156]. Cosmi et al. [133] assessed the predictive value of D dimer at 1 month after anticoagulation was stopped and residual venous obstruction in combination for recurrent VTE in a prospective cohort of patients after a first episode of idiopathic proximal DVT during a 2-year followup. The recurrence rate was 16.7% in all subjects, 7.9% in subjects with normal Ddimer, 24.9% in subjects with abnormal D dimer, and 14.9% and 18.2% in subjects without or with residual thrombus, respectively. The multivariate hazard ratio for recurrence was 3.32 (CI 1.78–6.75; *P* < 0.0001) for abnormal D dimer and 1.2 (CI 0.72–2.07) for presence of residual thrombus. These results indicate that increased D dimer at 30 days after VKA withdrawal is an independent risk factor for recurrent VTE; the persistence of residual thrombus at the time of VKA withdrawal in the presence of abnormal D dimer does not contribute to the risk of recurrence.

A prospective, collaborative, and randomized study (PROLONG study) was then performed to assess the predictive role of D dimer for recurrence after a first episode of unprovoked VTE [157]. The aims of the study were (a) to evaluate the safety of withholding VKAs in patients with normal D dimer assessed one month after VKAinterruption, (b) to establish the risk of recurrence in patients with altered D dimer at that time, and (c) to evaluate the protective effect of a resumption of VKA treatment in patients in the latter group. The same qualitative, fast method, performed on whole blood method for D dimer assay, was used in all centers. Patients with normal D dimer (n. 385) did not resume anticoagulation, whereas those with abnormal D dimer results were randomly assigned to either stop (n. 120) or resume (n. 103) VKAs. During the followup period of up to 18 months the incidence of recurrent VTE was 15.0% (10.9% patient-years) and 2.9% (2.0% patient-years) in the patients with abnormal D dimer randomized not to resume or to resume anticoagulation, respectively, and 6.2% (4.4% patient-years) in those with normal D dimer test. The followup of the Prolong study was extended to a mean of 2.55 years and confirmed the higher risk of recurrence associated with an abnormal postanticoagulation D dimer [158]. 

As expected, in the PROLONG study elderly patients had altered D dimer assay more often than the younger ones, and their hazard ratio was not statistically significant. The possible advantage of using quantitative D dimer assays with higher cutoff levels in patients aged >70 years was recently investigated by examining samples obtained from patients included in the PROLONG study, and a marked improvement of the predictive performance of D dimer assays in this subgroup of patients was found [159].

One meta-analysis [160] and one systematic review [161] of the available studies consistently confirmed that elevated D dimer levels measured 1 month after discontinuation of VKA treatment identify patients with idiopathic VTE who are at higher risk of recurrence. A patient-level meta-analysis [162] analyzed 1818 patients (from 7 studies) with a first unprovoked VTE, with D dimer measured after stopping anticoagulation, followed for a mean of 26.9 months, confirming that the risk for recurrent VTE was higher in patients with a positive D dimer result than in those with a negative result, regardless of the timing of postanticoagulation D dimer testing or patient age.

More recently, Cosmi et al., in the PROLONG II study [163], repeated the D dimer test every two months in patients who had normal D dimer one month after VKA withdrawal and found that in a small proportion of patients (12.7%) the test became abnormal at the third month and remained abnormal afterward and that these patients had a higher risk of recurrence (27% patient-years; CI 12–48) than patients with persistently normal D dimer (2.9%; CI: 1–7; adjusted HR: 7.9; CI: 2.1-30; *P* = 0.002). These results demonstrated that repeating the assay for the first 3 months after VKA interruption is useful to assess a late appearance of signs of hypercoagulability and to further identify subjects at higher risk of recurrence. 

### 6.2. D Dimer Measured during Anticoagulation

Some studies have investigated the value of D dimer measured during oral anticoagulation in patients with DVT. Fattorini et al. found that the probability of suffering recurrence was higher in patients whose D dimer levels were higher during and/or after anticoagulation withdrawal [164]. Rodger et al. measured D dimer levels during anticoagulation and found that values above a cutoff of 250 *μ*g/mL (using the Vidas D dimer reagent on the Vidas Instrument (bio-Mérieux)) for women, but not for men, were an important predictor of recurrence after the patient had stopped taking anticoagulants [64]. 

The predictive value for recurrent VTE of D dimer measured at hospital discharge in patients with acute PE was also addressed by a recent study, where a higher rate of recurrent VTE was found in those with abnormal assay at discharge (21%) compared to those with D dimer regression (6%; *P* = 0.001); persistently abnormal D dimer levels at discharge were an independent predictor of recurrent VTE (hazard ratio, 4.10; CI, 1.61–10.39; *P* = 0.003) [165].

### 6.3. Thrombin Generation Assay

A few studies have investigated the thrombin generation assay to assess the predictive value of the results for the risk of recurrent VTE. The results were, however, nonconcordant according to Hron et al. [166] the assay was able to identify patients at low or high risk for recurrence, but this result was not confirmed by another study [167]. More recently, Tripodi et al. [168] found that the measurement of thrombin generation may help identify patients at higher risk of VTE recurrence and that the test performs better if carried out in the presence of thrombomodulin.

## 7. Predicting the Risk of VTE Recurrence

Some of the abovementioned risk factors and putative predictors of thrombosis recurrence have been evaluated in order to be incorporated into clinical decision guides aiming at stratifying patients according to their individual high or low risk of recurrence; their final aim is to help clinicians decide about the duration of anticoagulation in the individual patient. Several guides have been proposed to this end, drawn from analyses of what happened in prospective cohorts of patients with previous unprovoked VTE. These risk assessments still require, however, validation in prospective cohorts of patients.

Rodger et al. [64] tried to identify patients at low risk of recurrent VTE, who could safely discontinue anticoagulation. They examined 69 potential predictors of recurrent VTE in a multicenter prospective cohort study of 600 patients with a first, unprovoked major VTE who completed a mean 18-month followup. The potential predictors were investigated while patients were under VKA treatment (5–7 months after initiation). While no combination of clinical predictors was found that satisfied criteria for identifying men at low risk, a low annual risk of recurrence (1.6%; CI 0.3–4.6), were found in women who had 0 or 1 of the following characteristics: hyperpigmentation, edema or redness of either leg; D dimer ≥250 *μ*g/L while taking warfarin; BMI ≥30 kg/m^2^; age ≥65 years. A high annual risk (14.1%; CI 10.9–17.3) was found in women who had 2 or more of the above characteristics. On the basis of these results and to guide decisions for the duration of anticoagulant treatment the authors proposed a clinical decision rule called “*Men continue and HER DOO2,*” where HER stands for hyperpigmentation, edema, or redness; DOO for: Vidas D dimer ≥250 *μ*g/L, obesity (BMI ≥30 kg/m^2^), old age (≥65 years); 2 for: less (low risk) or more (high risk) than 2 of the above characteristics.

Eichinger et al. [55], by analyzing a prospective cohort of more than 900 patients with a first unprovoked VTE followed up for a median of 43.3 months after discontinuation of anticoagulation, computed a *nomogram* to be used to calculate risk scores and to estimate the cumulative probability of recurrence in individual patients. The nomogram is based on the combination of three variables that in the examined cohort were significantly (at Cox-regression analysis) associated with recurrence. The variables related to a higher recurrence risk were sex (male > female), location of first thrombosis (PE > proximal DVT > distal DVT) and elevated levels of D dimer measured afteranticoagulation (used as a continuous variable).

Tosetto et al. [169] performed a meta-analysis of individual data of 1818 patients drawn from prospective studies that included patients with a first unprovoked VTE who had received anticoagulant therapy for at least 3 months and were followed for up to 5 years after treatment was stopped. They found that abnormal D dimer after stopping anticoagulation, age < 50 years, and male sex were conditions associated with higher risk of recurrence, whereas VTE associated with hormonal therapy (in women) was associated with lower risk. They then elaborated a prognostic recurrence score called “*DASH*,” that included D dimer (2 points), age (1 point), sex (male 1 point), and hormonal therapy (−2 points). By applying the DASH score the annualized recurrence risk was 3.1% (CI, 2.3–3.9) in subjects with a score ≤1; 6.4% (CI, 4.8–7.9) when the score was = 2 and 12.3% (CI, 9.9–14.7) for scores ≥3. On the basis of the low recurrence risk for patients with a score ≤1, the authors suggested avoiding life-long anticoagulation in these patients, that were about half of all patients included in the study.

## 8. Current Recommendations and Guidance on Duration of Anticoagulant Therapy after a First Unprovoked VTE

In the last edition (9th) of the Antithrombotic Therapy and Prevention of Thrombosis ACCP Guidelines, Kearon et al. [50] recommend patients with an unprovoked DVT or PE be treated with anticoagulation for at least 3 months (Grade 1B), after which each patient should be evaluated for the risk-benefit ratio of extended therapy, where the term “extended” means continued anticoagulation without a scheduled stop date, but with periodic reassessments to check if the patient's bleeding risk is not increased and his preference not changed (Grade 2B).

Similarly, recent guidance from the SSC of the ISTH [170] concluded that patients with a first or recurrent episode of unprovoked PE or proximal DVT should be considered for long-term anticoagulation since their annual risk of recurrence is >5%, a rate that exceeds the risk of VKA-related bleeding.

### 8.1. Risk and Consequences of VKA-Related Bleeding

In unselected patients who started VKA anticoagulation for the first time for whatever indication it was shown that the rate of bleeding (major and minor) was significantly higher during the first 3 months of treatment than thereafter [171]. This finding was confirmed more recently by Linkins et al. [172], who in a meta-analysis of available studies found a 2% risk of major bleeding during the first 3 months of treatment in patients treated for VTE, and a 2.7% patient-years in the subsequent period. The rate of intracranial hemorrhage, the most feared complication during anticoagulation, was 1.48% patient-years during the initial 3 months of therapy but decreased to 0.65% patient-years thereafter. 

Many factors (Table 2) may influence the risk of bleeding during VKA treatment (for a review on this issue see [173]). On the basis of presence of risk factors Kearon et al. [50] suggest the following categorization of risk of bleeding in anticoagulated patients: (i) low (estimated absolute risk of major bleeding of 0.8%) in patients without risk factors, (ii) moderate (1.6%) in those with 1 factor, and (iii) high (≥6.5%) in those with ≥2 factors.

Treatment- and person-associated factors have been combined in clinical prediction rules in an attempt to identify those patients at a higher risk of bleeding during anticoagulation. The aim of these prediction rules is to help physicians stratify patients into categories of risk and evaluate the risk/benefit ratio of anticoagulation upon starting and prolonging this treatment. To date several clinical prediction rules have been proposed (see [173]). The recently proposed HAS-BLED score [174] (Table 3) not only gives a global assessment of bleeding risk of a patient but also gives to the clinician indication regarding the possible risk factors that can be modified during anticoagulation (e.g., a more accurate control of anticoagulation or of hypertension, or withdrawal of aspirin administration if associated). Unfortunately, the HAS-BLED score has so far been validated in patients with atrial fibrillation [175] but not in patients treated for venous thromboembolism. 

 The risks of major bleeding during extended VKA treatment can be estimated in a range of 0.9–3.0% per year on the basis of the results of trials on duration of anticoagulation [12, 102, 103, 105, 106]. It is worth noting, however, that the duration of observation in these anticoagulation trials is relatively short (only 2-3 years) and that the included patient population is usually selected. 

 A major bleeding complication may be life threatening and, in any case, may cause significant clinical sequelae. It is important to analyze the case-fatality rate of major bleeding to weigh the risk of extended anticoagulation versus the risk of recurrent VTE in cases where anticoagulation is interrupted. Linkins et al. [172] analyzed clinical studies involving patients receiving VKA for VTE and found that the case-fatality rate of major bleeding in those treated for more than 3 months was 9.1% (CI, 2.5–21.7), and the rate of intracranial bleeding was 0.65% patient-years (CI, 0.63–0.68) after the initial 3 months of anticoagulation. They concluded that the clinical impact of anticoagulant-related major bleeding in patients with VTE is considerable and should be taken into account when deciding whether to continue long-term VKA therapy. Punthakee et al. [176] found a case fatality rate of 45% (CI, 23–67) in patients with intracerebral hemorrhage while receiving VKA therapy. More recently, Carrier et al. [37], in a systematic review of prospective cohort studies and randomized controlled trials involving patients treated for VTE, assessed the case-fatality rates of major bleeding events during anticoagulation (for at least 3 months) and recurrent VTE after anticoagulation. They found that during anticoagulation the rate of fatal major bleeding events was 0.2% (CI, 0.1–0.3), with a case-fatality rate of 11.3% (CI, 7.5–15.9), while after anticoagulation the rate of fatal recurrent VTE was 0.3% patient-years (CI, 0.1–0.4), with a case-fatality rate of 3.6% (CI, 1.9–5.7). These data point up the higher risk of mortality associated with a major bleeding event during anticoagulation than that associated with recurrent VTE after anticoagulation is stopped.

## 9. Practical Indications on How to Manage Patients with DVT or PE

For some kinds of patients the decision for management of anticoagulation is relatively easy. This is the case for conditions at low risk of recurrence, such as distal DVT and VTE events secondary to a transient trigger factor where a short period of anticoagulation (usually from 3 to 6 months) is sufficient. Conversely, there is agreement on the need for indefinite anticoagulant treatment in conditions that are known to be at high risk of recurrence, such as cancer-associated VTE, antiphospholipid syndrome, second unprovoked VTE, or multiple thrombotic events, presence of strong prothrombotic thrombophilic alterations.  Table 4, similar—though with some differences—to the one proposed by Schulman and ögren [177], shows in some detail the suggested management options in different clinical conditions. 

More uncertain is the best management for patients with a first unprovoked event, a condition that involves a large portion of all VTE patients. In these patients the estimated cumulative risk of recurrent VTE after stopping anticoagulant therapy is high (10% recurrence after 1 year, 30% after 5 years, and less than 50% at 10 years). This has prompted guidelines to suggest an extended anticoagulation in patients with unprovoked events, provided that they can perform well managed anticoagulation, have low risk of bleeding, and their conditions are periodically reassessed [50]. Some physicians are reluctant to treat all such patients indefinitely, and some authors are explicitly against this general recommendation [178]. First of all, the risk of recurrent events gets lower over the years, while that of VKA-related bleeding is expected to be stable after the first 3 months of treatment. The randomized controlled trials were probably not long enough to assess the risk/benefit relation of chronic anticoagulation over a very long period. Finally, since it is expected that only about half of these patients will have a recurrent VTE in 10 year's time, it does not seem clinically justified to give indefinite anticoagulation, with the associated risks and burden for patients and health systems, to all the patients, including the 50% of them that would not have had a recurrence in any case. 

### 9.1. Different Strategies for Secondary Prevention

The use of extended VKA—anticoagulation at low-intensity (INR 1.5–2.0) after an initial treatment of 3–6 months of conventional—intensity anticoagulation (INR 2.0–3.0) was put forward as a way of reducing the risk of associated bleeding events and improving the quality of life of patients and was found to be more efficacious than placebo [105]. A second randomized controlled study showed, however, that the low-intensity regimen was less efficacious versus the standard regimen without any advantage in terms of safety [106].

A recent multicenter, double-blind study (WARFASA) randomly assigned to aspirin (100 mg daily, or placebo for 2 years) patients with first unprovoked VTE after they had completed 6 to 18 months of VKA treatment [179]. Recurrent VTE occurred in 28/205 patients who received aspirin and in 43/197 patients who received placebo (6.6% versus 11.2% per year; HR, 0.58; CI, 0.36 to 0.93); one patient in each treatment group had a major bleeding episode. The authors conclude that aspirin reduces the risk of recurrence in patients with unprovoked VTE after discontinuation of anticoagulant treatment, with no apparent increase in the risk of major bleeding.

Different strategies are based on a switch from a disease-focused approach (all patients with unprovoked VTE should be treated long term in the same way) to individual patient-focused management decisions, by trying to stratify the individual risk of recurrence and give individual advise. A recent official communication of the SSC of ISTH [180] recommended as a clinical priority the need for studies focused on identification of subgroups of patients with unprovoked proximal DVT or PE who have a low risk of recurrence; necessary following steps are validation studies showing that it is safe to stop anticoagulation in these subgroups of patients. A further problem is deciding on the level of risk of recurrent VTE that is acceptable after stopping anticoagulant therapy for VTE. In this regard, the same SSC official communication [180] puts forward as acceptable the risk of recurrence that prevails after 3 months of anticoagulant therapy in patients with VTE provoked by a nonsurgical transient provoking factor, estimated at about 5% at 1 year and 15% at 5 years [48], and gives detailed recommendations for cohort studies to tackle this issue.

The risk assessment schemes already mentioned for predicting the individual risk of VTE recurrence [55, 64, 169] have still not been tested in prospective clinical studies. 

### 9.2. The DULCIS Study

A prospective, multicenter management study in a cohort of patients with unprovoked first-ever VTE (the DULCIS study, D dimer and Ultrasonography in Combination Italian Study, ClinicalTrials.gov NCT00954395) closed in May this year, and analysis of results is underway. In synthesis, following inclusion and exclusion criteria, the study included patients with proximal DVT and/or PE. After a standard period of VKA anticoagulation (in average about 6 months) patients were evaluated for possible VKA interruption. A deep vein ultrasonography examination was performed in all patients, and a period of one year VKA therapy was recommended for those patients with significant residual vein obstruction (>4 mm diameter at full compression). Patients with PE, if without signs of pulmonary hypertension, those without residual vein obstruction or who had been treated with VKA for at least one year for the presence of residual vein obstruction, received D dimer testing the day they were examined for anticoagulation withdrawal and at intervals during the first 3 months after VKA suspension. Whenever the results of D dimer testing were above prefixed cutoff values—specifically established in relation to method used, sex, and age—continuation or resumption of anticoagulation was recommended. The pre-specified targets of the study were to allow interruption of anticoagulation in at least 40% of patients included and to have a rate of VTE recurrence not >5% per year in these patients.

## Figures and Tables

**Table 1:** Risk factors for recurrence after a first VTE event.

Thrombosis related	Patient related
Unprovoked event Nonsurgical transient versus surgical risk factor associated with the first event Proximal (especially if iliofemoral) versus distal DVT Pulmonary embolism Persistence of residual vein thrombosis	Men Active cancer Antiphospholipid syndrome Inherited thrombophilic alterations Pregnancy and puerperium Hormonal therapies Obesity Presence of inferior vena cava filter Polycythemia vera and essential thrombocythemia, especially in the presence of JAK2 V617F mutation

**Table 2:** Factors that increase the risk of VKA-related bleeding.

(A) Treatment-associated factors	
First 3 months of therapy	
Target intensity of anticoagulation > 2.0–3.0 INR	
Actual INR values > 4.5	
Low quality of anticoagulation monitoring (high % time out of range)	
Use of short half-life VKA drug	
(B) Person-dependent factors	
Advanced age (>75 years)	
Frequent falls	
History of major bleeding (especially in the GI tract)	
History of atherosclerotic stroke	
Uncontrolled hypertension	
Cancer	
Congestive heart failure	
Anemia	
Renal or liver failure	
Alcohol abuse	
Recent surgery	
Associated antiplatelet therapy	
Frequent use of NSAIDs	
Polymorphisms of VKORC1 and CYP2C9	
Mutation in factor IX propeptide (low factor IX levels)	
Drugs affecting pharmacokinetics or pharmacodynamics of VKAs	
Insufficient information and education to the treatment	
Poor compliance	
Poor dietary intake of vitamin K	
Nutritional supplements and herbal products	
Absence of familial or social support	

**Table 3:** The HAS-BLED score for the assessment of bleeding risk in patients treated with warfarin.

Risk factors	Score for each risk factor	Total score	Major bleeding events (% patients) in relation to the total score
None	/	0	0.9
Hypertension	1	1	3.4
Abnormal renal or liver function	1 each	2	4.1
Stroke	1	3	5.8
Bleeding history or predisposition	1	4	8.9
Labile INR	1	5	9.1
Age > 65 years	1	6	0
Drugs/alcohol concomitantly	1 each	/	/

(From Lip et al. modified [175]).

**Table 4:** Suggested management of anticoagulation (AC) for secondary prophylaxis in patients with VTE (DVT and/or PE).

Clinical condition	Management
Secondary* isolated distal DVT	6 weeks AC^#^
Unprovoked isolated distal DVT	3 months AC
Secondary proximal DVT and/or PE	3–6 months AC
Unprovoked first proximal DVT and/or PE	3–6 months AC, then stratify for individual risk of recurrence
Life-threatening PE as index event	Consider extended^*£*^ AC
VTE associated to active cancer	AC until cancer is no longer active
Unprovoked VTE associated with antiphospholipid syndrome	Consider extended AC
Unprovoked VTE associated with antithrombin, deficiency	Extended AC
Unprovoked VTE associated with other major thrombophilic alteration (protein C or S deficiency, homozygous factor V Leiden or G20210A prothrombin mutation or double heterozygous)	Consider extended AC
Second unprovoked VTE	Extended AC
Third VTE	Extended AC

*Secondary when associated with one of the following triggering factors: major surgery, serious trauma, immobilization, bed resting for >4 days, pregnancy, and puerperium.

^#^Consider treatment with LMWH.

^*£*^Extended: a continueous anticoagulation without a scheduled stop date, but with periodic reassessments to verify that the patient's bleeding risk is not increased and his preference is not changed.
